# Temporal dynamics of gait function in acute cervical spinal cord injury

**DOI:** 10.1186/s12891-024-07551-6

**Published:** 2024-06-03

**Authors:** Hiroki Okayasu, Tetsuo Hayashi, Kazuya Yokota, Osamu Kawano, Hiroaki Sakai, Yuichiro Morishita, Muneaki Masuda, Kensuke Kubota, Hiroshi Ito, Takeshi Maeda

**Affiliations:** 1 Department of Orthopaedic Surgery, Japan Organization of Occupational Health and Safety, Spinal Injuries Center, Fukuoka, Japan; 2 Department of Orthopaedic Surgery, Asahikawa Medical University, 2-1-1-1, Midorigaoka Higashi, Asahikawa, Hokkaido 78-8510 Japan; 3 Department of Rehabilitation Medicine, Japan Organization of Occupational Health and Safety, Spinal Injuries Center, Fukuoka, Japan

**Keywords:** Cervical spinal cord injury, Gait function, Time course

## Abstract

**Background:**

Following spinal cord injury (SCI), gait function reaches a post-recovery plateau that depends on the paralysis severity. However, the plateau dynamics during the recovery period are not known. This study aimed to examine the gait function temporal dynamics after traumatic cervical SCI (CSCI) based on paralysis severity.

**Methods:**

This retrospective cohort study included 122 patients with traumatic CSCI admitted to a single specialized facility within 2 weeks after injury. The Walking Index for Spinal Cord Injury II (WISCI II) was estimated at 2 weeks and 2, 4, 6, and 8 months postinjury for each American Spinal Injury Association Impairment Scale (AIS) grade, as determined 2 weeks postinjury. Statistical analysis was performed at 2 weeks to 2 months, 2–4 months, 4–6 months, and 6–8 months, and the time at which no significant difference was observed was considered the time at which the gait function reached a plateau.

**Results:**

In the AIS grade A and B groups, no significant differences were observed at any time point, while in the AIS grade C group, the mean WISCI II values continued to significantly increase up to 6 months. In the AIS grade D group, the improvement in gait function was significant during the entire observation period.

**Conclusions:**

The plateau in gait function recovery was reached at 2 weeks postinjury in the AIS grade A and B groups and at 6 months in the AIS grade C group.

## Background

Spinal cord injury (SCI) is a major cause of severe motor impairments [1]. In addition to causing walking difficulties, it has physical (circulatory dynamics) [2], psychological [3], and even financial effects [4]. Various therapeutic interventions for SCI have been attempted, and accurately predicting the functional prognosis is necessary to determine treatment effectiveness. SCI functional prognosis depends on the paralysis severity at the time of injury [5], with a slight improvement in complete injuries [6] and comparatively good improvement in incomplete injuries [7]. Most functional prognosis studies have used the American Spinal Cord Injury Association Impairment Scale (AIS) grade or motor score [5, 7]. However, these indicators are based on muscle strength assessments and do not directly reflect the ability to perform activities of daily living (ADLs). This distinction is important because cases vary in clinical practice, including cases of severe paralysis with maintained ADLs and cases of mild paralysis with poor ADLs.

Among ADLs, gait function has a significant impact on patient well-being [8]. The Walking Index for Spinal Cord Injury II (WISCI II) has been used to assess gait function after injury [9–12] in patients with SCI by evaluating their motor functions using a scale of 0 to 20, based on the types of the orthotic device and walking aid used and the degree of physical assistance provided: the lower the value, the lower the motor function [10]. The WISCI II system has excellent criteria-based validity, reliability, and sensitivity to change, and it is regularly used for gait function assessments [12]. However, few longitudinal studies on gait function have been conducted in patients with SCI.

As with other motor functions, gait function in patients with SCI reaches a plateau in recovery based on the paralysis severity [13]. A statistical evaluation of the recovery plateau has not yet been reported, and identifying the plateau timing could help infer the rehabilitation goal. Additionally, the timing of reaching a plateau is believed to reflect the rehabilitation effectiveness and spontaneous recovery limits, which are important factors for determining the intervention’s optimal timing in clinical trials for novel therapies, such as robotic rehabilitation and regenerative medicine. Therefore, this study aimed to clarify the temporal dynamics of gait function in patients with acute cervical SCI (CSCI) based on paralysis severity.

## Methods

### Study design and participants

Patients with traumatic CSCI (neurological level of injury C1, C2, …, C8) who were admitted to our hospital within 14 days after injury between October 2013 and September 2021 were included. We excluded patients with a follow-up duration shorter than 8 months and those who could not be evaluated at the appropriate time points (e.g., patients with impaired consciousness or dementia, those with deteriorated general condition, patients discharged early owing to a marked improvement in their paralysis or ADL impairment, etc.). Our hospital is a specialized facility for SCIs and provides support from the hyperacute stage immediately after injury to outpatient follow-up after reintegration into society. The severity of hospitalized patients varies from severely ill patients who require long-term inpatient rehabilitation to mildly ill patients who are able to return to society relatively quicker. All patients in this study underwent rehabilitation at our hospital.

Patient data were retrospectively reviewed using the Japan Single-Center Study for Spinal Cord Injury Database [14]. Eligible patients were classified based on the AIS grade determined 2 weeks after injury. The physical examination, determination of AIS grade, and WISCI II evaluation were supervised by a qualified International Standards Training e-Learning Program (InSTeP) expert familiar with SCIs.

The Institutional Review Board of the Spinal Injuries Center (Fukuoka, Japan) approved the study (approval number 18 − 5), and all participants provided written informed consent before participating.

### Evaluation and statistical analysis

WISCI II scores at 2 weeks and 2, 4, 6, and 8 months postinjury were evaluated for each AIS grade (as determined 2 weeks postinjury). The analysis was performed at 2 weeks to 2 months, 2–4 months, 4–6 months, and 6–8 months; the time at which no significant difference was observed was considered the time the gait function reached a plateau. The Wilcoxon signed rank-sum test was used for statistical analysis, and a p-value < 0.05 was considered statistically significant. Statistical analysis was performed using Statcel 4 software (OMS Publishing Inc., Saitama, Japan).

## Results

Of the 128 patients, we excluded 6 who were not evaluated for gait function due to deterioration in their general condition or because they required a transfer for treatment (sepsis [*n* = 2], ileus [*n* = 1], gastrointestinal bleeding [*n* = 1], hyperkalemia [*n* = 1], and participating in a trial at another hospital [*n* = 1]). In total, 43, 24, 28, and 27 patients were classified into AIS grade A, B, C, and D groups, respectively. The patients’ demographics are shown in Table 1.

**Table 1:** Patients’ demographic data (*n* = 122)

Age (years)	58.3 ± 16.3
**Sex**	
Women	19 (15.6%)
Men	103 (84.4%)
**AIS grade**	
A	43 (35.2%)
B	24 (19.7%)
C	28 (23.0%)
D	27 (22.1%)
**Neurological level of injury**	
C1	0 (0%)
C2	12 (9.8%)
C3	17 (13.9%)
C4	55 (45.1%)
C5	23 (18.9%)
C6	10 (8.2%)
C7	3 (2.5%)
C8	2 (1.6%)

Age is expressed as mean ± standard deviation

AIS, American Spinal Cord Injury Association Impairment Scale

**Fig. 1:**
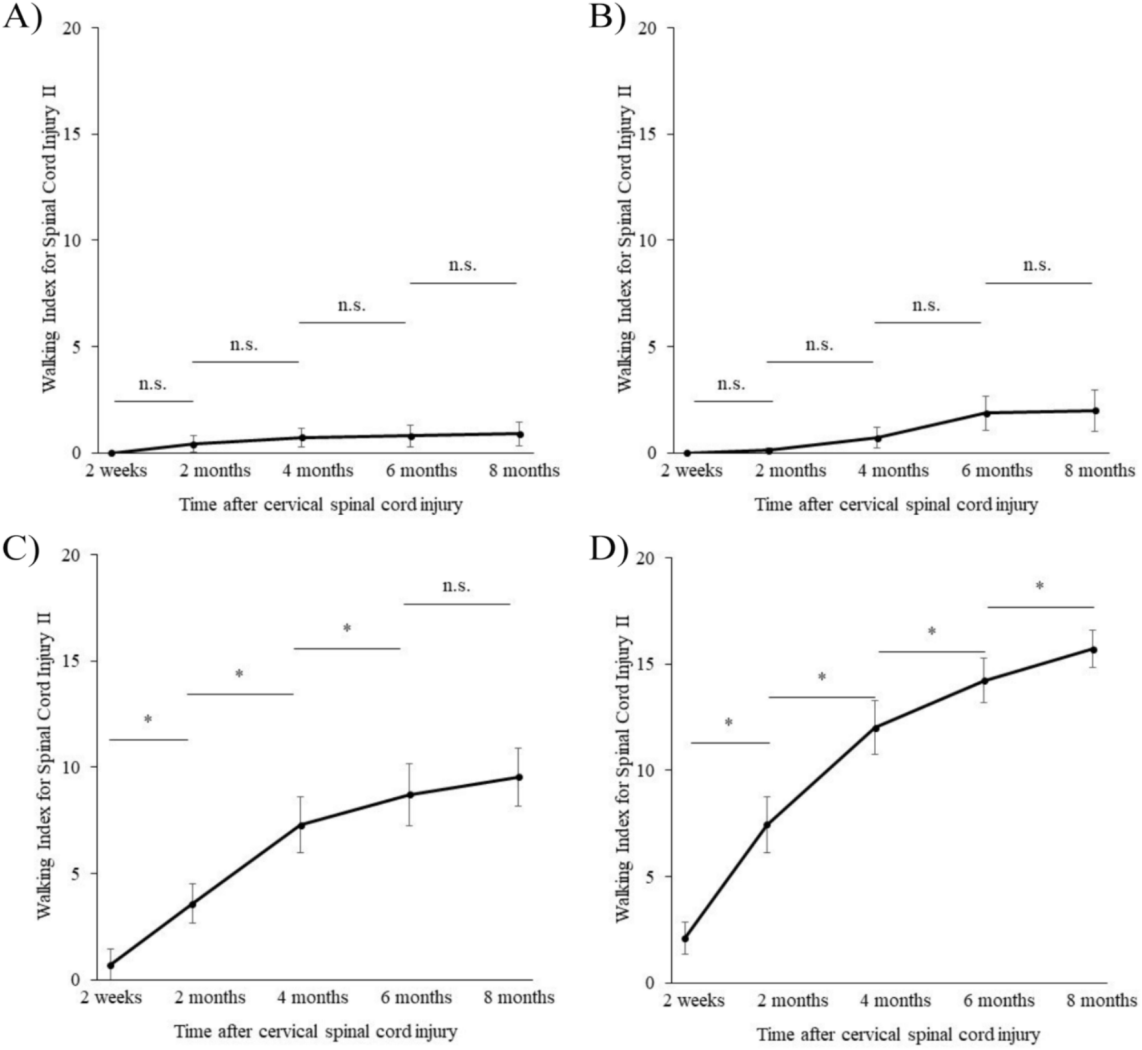
Changes in the Walking Index for Spinal Cord Injury II (WISCI II) over time. Mean values for each American Spinal Cord Injury Association Impairment Scale (AIS) grade determined at 2 weeks after injury (**A**, AIS grade A; **B**, AIS grade B; **C**, AIS grade C; **D**, AIS grade D). The Wilcoxon signed rank-sum test was used for statistical analysis, and a P-value < 0.05 was considered statistically significant. n.s., not significant; * P-value < 0.05

In the AIS grade A and B groups, some patients showed improved WISCI II scores with higher mean values, but the differences were not statistically significant (Fig. 1-A and -B, Table 2). The gait function plateaued at 2 weeks postinjury, and the median WISCI II score was 0 during the entire observation period (Table 2). In contrast, the mean WISCI II values continued to increase in the AIS grade C group, with a significant improvement up to 6 months (Fig. 1-C, Table 2), indicating the gait function plateau moment. Finally, in the AIS grade D group, both mean and median WISCI II values increased, and the gait function continued to significantly improve throughout the entire observation period (Fig. 1-D, Table 2).

**Table 2:** Walking index for spinal cord injury II trends and statistics

	Time after cervical spinal cord injury				
	2 weeks	2 months	4 months	6 months	8 months
**AIS grade A (*n*** **=** **43)**					
Median (IQR)	0 (0–0)	0 (0–0)	0 (0–0)	0 (0–0)	0 (0–0)
Average ± SE	0 ± 0	0.42 ± 0.40	0.72 ± 0.45	0.79 ± 0.51	0.91 ± 0.56
P-value		n.s.	n.s.	n.s.	n.s.
**AIS grade B (*n*** **=** **24)**					
Median (IQR)	0 (0–0)	0 (0–0)	0 (0–0)	0 (0–0)	0 (0–0)
Average ± SE	0 ± 0	0.21 ± 0.13	0.71 ± 0.49	1.88 ± 0.80	2.00 ± 0.98
P-value		n.s.	n.s.	n.s.	n.s.
**AIS grade C (*n*** **=** **28)**					
Median (IQR)	0 (0–0)	0 (0–8)	8 (0–13.25)	9.5 (0–17)	8 (2–17)
Average ± SE	0.71 ± 0.71	3.57 ± 0.92	7.29 ± 1.33	8.71 ± 1.45	9.54 ± 1.37
P-value		0.01*	0.00*	0.04*	n.s.
**AIS grade D (*n*** **=** **27)**					
Median (IQR)	0 (0–2)	8 (0–13.8)	14 (8–17)	16 (9–19.5)	17 (14–20)
Average ± SE	2.11 ± 0.74	7.44 ± 0.77	12.00 ± 1.32	14.22 ± 1.04	15.7 ± 0.89
P-value		< 0.01*	< 0.01*	0.01*	0.02*

P-values show statistically significant values compared to the previous measurements

AIS, American Spinal Cord Injury Association Impairment Scale, IQR; interquartile range, SE; standard error, n.s.; not significant

* P-value < 0.05

## Discussion

In this study, we investigated the temporal dynamics of the gait function in patients with traumatic CSCI based on the AIS grades evaluated at 2 weeks postinjury using the WISCI II system. In patients with AIS grade C, the gait function reached a plateau after 6 months. This is an important criterion when considering the targets and timing of clinical trials for testing new treatments.

The AIS grade A and B groups exhibited no significant improvement in gait function at 2 weeks postinjury. A previous study reported that the plateau in AIS grade A injuries was achieved 1 week postinjury, and 80–90% of patients remained at AIS grade A [6, 15, 16]. Previously, a modest improvement in paralysis symptoms was reported 2 weeks postinjury, although the degree was low [17]. The lack of muscle strength improvement in these groups could also explain the lack of improvement in gait function. Therefore, in these groups, it would be more realistic to focus on other functional recovery measures, such as improving upper limb function and adjustment to the environment, rather than on gaining the ability to walk. In contrast, the gait function in the AIS grade D group continued to significantly improve during the observation period, and it was expected to improve even with long-term gait training using the conventional approach.

In patients with SCI, the Spinal Cord Independence Measure III and Functional Independence Measure are commonly-used tools to assess patients’ ability to perform ADLs. However, these tools evaluate only overall ADLs, while an evaluation of gait function is simpler. As an assessment specific to gait function, the WISCI II has an established reputation for excellent criterion-based validity, reliability, and sensitivity to change [12]. In this study, we used WISCI II to assess gait function with the caveat of considering regional differences in the guidelines for using orthotics and gait aids [18] and the training needed to perform the assessment [11]. WISCI II was developed for a typical North American walking aid and may not be ideal for a typical Asian or European walking aid. This study was conducted at a single site, and thus the results were not influenced by regional differences. Proper evaluation of WISCI II requires examination by an experienced and trained clinician, and to our knowledge, there are no specific qualifications for WISCI II. SCI treatment experts, including InSTeP-certified individuals, provided guidance on WISCI II assessment, and its validity was considered appropriate. Therefore, the accuracy of the physical examination and scoring is considered guaranteed in our study.

This study has several limitations. First, only patients who were followed up for more than 8 months were included. As also noted in previous studies [19], the AIS grade D group had a shorter follow-up duration than those of the other groups due to rapid functional recovery. This introduced a bias that might have led to an underestimation of the recovery rate, particularly in the AIS grade D group. Additional studies are needed to elaborate the walking ability in this group. Second, it has been suggested that the WISCI II system should be used in combination with the 10-meter walking test due to the ceiling effect, and not by itself [12]. Indeed, WISCI II cannot evaluate some functions, and its combination with other measures, such as speed, endurance, and balance, may help assess plateaus in walking function from a multifaceted perspective. Although only a small number of patients in the current study scored 20 on the WISCI II scale, suggesting a low impact of the ceiling effect, the study’s validity might have been enhanced if used in conjunction with other measures. Finally, the definitive timing of the post-recovery plateau cannot be specified based on the statistical methods used in this study. To provide a clear and reliable assessment, it is necessary to clarify whether subtle improvements in the chronic phase are clinically meaningful [20]. Accordingly, future studies should use additional tests to ensure that definitive findings are reached.

The results of this study could help improve gait function based on paralysis severity. Spontaneous recovery is often an issue in clinical trials of novel therapies, and to minimize its impact, it may be necessary to intervene in the chronic phase after the gait function has reached a plateau. In clinical trials for improving gait function after traumatic CSCI, the novel treatment’s effectiveness could be more evident if the intervention in the AIS group C is initiated 2 weeks, 6 months, or later after injury.

In conclusion, the gait function of patients with AIS grade A and B injuries plateaued at 2 weeks postinjury, whereas that of patients with AIS grade C injuries plateaued at 6 months postinjury.

## Data Availability

The datasets used and/or analysed during the current study available from the corresponding author on reasonable request.

## Abbreviations

SCI: Spinal cord injury
CSCI: Cervical SCI
WISCI II: Walking Index for Spinal Cord Injury II
AIS: American Spinal Injury Association Impairment Scale
ADLs: Activities of daily living
InSTeP: International Standards Training e-Learning Program
